# Retrospective analysis of seven cases of pancreatic mixed adenoneuroendocrine carcinoma from a high-volume center and review of the literature

**DOI:** 10.1186/s12893-019-0546-0

**Published:** 2019-07-11

**Authors:** Feng Tian, Meng-Hua Dai, Cong-Wei Jia, Zi-Wen Liu, Bing-Lu Li

**Affiliations:** 1 Department of General Surgery, Peking Union Medical College Hospital, Chinese Academy of Medical Sciences and Peking Union Medical College, No. 1, Shuaifuyuan, Wangfujing Avenue, Dongcheng District, Beijing, 100730 China; 2 Department of Pathology, Peking Union Medical College Hospital, Chinese Academy of Medical Sciences and Peking Union Medical College, Beijing, 100730 China

**Keywords:** Clinicopathological, Pancreatic, Mixed adenoneuroendocrine carcinoma, Survival

## Abstract

**Backgrounds:**

The clinicopathologic features and biological behaviors of pancreatic mixed adenoneuroendocrine carcinoma (pMANEC) and its impacts on survival are poorly known.

**Methods:**

We retrospectively reviewed seven pMANEC cases from a single institution from September 2010 to January 2017 along with twenty-one previously reported cases from the literature. Survival and prognostic analyses were conducted using Kaplan-Meier estimates and Cox regression, respectively.

**Results:**

Seven pMANEC cases were identified during the study interval. Among the six patients who underwent operations, five reached R0 resections, one experienced postoperative pancreatic fistula, and two suffered other complications. The median progression-free survival (PFS) and disease-specific survival (DSS) were 7.5 months (2 to 36 months) and 15 months (6 to 36 months), respectively. A total analysis of twenty-eight pMANEC cases showed that patients were mostly older (median age, 59.5 years) and male (64.3%). The two most common symptoms were abdominal pain (53.6%) and obstructive jaundice (35.7%). The majority of pMANECs were non-functional (89.3%) and located in the pancreatic head (64.3%). The median diameter of pMANEC was 3.0 cm, with a wide range (0.5 to 19.0 cm). Lymph node metastasis (*P* = 0.015) was associated with decreased DSS, while age (*P* = 0.414), sex (*P* = 0.125), tumor size (*P* = 0.392), location (*P* = 0.913), functional status (*P* = 0.313), CA19–9 level (*P* = 0.608), and liver metastasis (*P* = 0.935) did not show significant prognoses on DSS.

**Conclusions:**

We reported seven pMANEC cases and outlined their clinical behaviors and prognoses with a review of twenty-one cases from the literature. Lymph node metastasis was found to be a negative prognostic factor of DSS based on the present study.

## Background

Pancreatic Mixed adenoneuroendocrine carcinoma (pMANEC) is a mixture of ductal and endocrine tumor cells, each component comprises at least 30% of the tumor tissue according to the 2010 WHO classification of digestive disease [1]. pMANEC is an extremely rare entity and has been reported to account for only 0.2% of all pancreatic tumors [2].

pMANEC remains mysterious due to the lack of literature available. Previously reported cases have various disease procedures and outcomes. To date, the clinicopathologic features and long-term postoperative course of the disease remain unclear due to its low prevalence. Neither the role of radical resection nor the effect of adjuvant radiotherapy and/or chemotherapy is clear. Further investigations on the biological behavior of pMANEC and how it differs from PDAC only or pNET only would increase knowledge about the origin and histogenesis of pancreatic tumors.

Here, we report seven pMANEC cases from a single institution from September 2010 to January 2017 and analyze the survival data of a combined cohort with twenty-eight pMANEC cases.

## Methods

### Patient selection and data acquisition

Patients diagnosed with pancreatic tumors (both malignant or benign) via biopsy or surgical resection at a single institution from September 2010 to January 2017 were reviewed. Cases were included if pMANEC was diagnosed by a pathological approach. Clinicopathological data were reviewed retrospectively based on the medical-record database. The data extracted include basic information (such as gender, age, symptoms, function status, tumor marker level and radiologic evaluation), surgery-related variables (surgical procedure, operative time, estimated blood loss (EBL), transfusion, R0 resection, postoperative pancreatic fistulae (POPF) and other complications, postoperative length of hospital stay (LOS)), and histopathological variables (tumor location, tumor size, Ki-67 index, WHO classification, lymph node (LN) metastasis, distal metastasis, vascular invasion, nerve invasion and fat infiltration). This study was approved by the ethics committee of Peking Union Medical College Hospital. All patients provided written informed consent.

An English literature search was performed for studies published before January 31, 2017 with the following strategy: ((((((((Malignant Mixed Tumor) OR (Malignant Mixed Tumors) OR (Mixed Tumors, Malignant) OR (Tumor, Malignant Mixed) OR (Tumors, Malignant Mixed))) OR “Mixed Tumor, Malignant”[Mesh])) OR mixed adenoneuroendocrine tumor)) AND ((((Neoplasm, Pancreatic) OR (Pancreatic Neoplasm) OR (Pancreas Neoplasms) OR (Neoplasm, Pancreas) OR (Neoplasms, Pancreas) OR (Neoplasms, Pancreatic) OR (Cancer of Pancreas) OR (Pancreas Cancers) OR (Pancreas Cancer) OR (Cancer, Pancreas) OR (Cancers, Pancreas) OR (Pancreatic Cancer) OR (Cancer, Pancreatic) OR (Cancers, Pancreatic) OR (Pancreatic Cancers) OR (Cancer of the Pancreas) OR (Disease, Pancreatic) OR (Diseases, Pancreatic) OR (Pancreatic Disease))) OR (“Pancreatic Diseases”[Mesh] OR “Pancreas”[Mesh] OR “Pancreatic cancer, adult” [Supplementary Concept] OR “Pancreatic Neoplasms”[Mesh]))). Titles, abstracts, and subsequently full-text articles were screened by two persons independently. Papers reporting pathologically diagnosed pancreatic mixed adenoneuroendocrine tumor were extracted and the pathological diagnoses were confirmed by a pathologist. The references of all included papers and PubMed ‘related articles’ were screened manually to identify initially missed but relevant studies. We reached the final decision on eligibility through intensive discussion and a total of twenty-one previously reported pMANEC cases were identified.

### Definitions

The postoperative courses for all patients were reviewed carefully to appropriately assess tumor characteristics and patient survival. R0 resection indicated a complete resection with microscopically negative margins (including retroperitoneal margin), whereas non-R0 resection indicated microscopically positive margins (R1 resection) or macroscopically residual disease (R2 resection). Operative time was calculated as skin-to-skin time. The POPF rate was re-classified based on the 2016 version of the International Study Group of Pancreatic Fistula (ISGPF) criteria [3]. Tumor grading was re-assessed based on the 2017 WHO-AJCC grading system for pancreatic neuroendocrine tumors: NET G1, Ki-67 index of 2% or less and mitotic rate of 1 or less per 10 high-power fields (HPF); NET G2, Ki-67 index above 2% but no more than 20% and mitotic rate greater than 1 but no more than 20 per 10 HPF; NET or NEC G3, Ki-67 index more than 20% and mitotic rate greater than 20 per 10 HPF [4]. MANEC was defined as a neoplasm with dual adenocarcinomatous and neuroendocrine differentiation, and each component accounted for at least 30% of the tumor [1]. However, amphicrine tumors refer to one cell type harboring both exocrine and endocrine markers simultaneously. The collision type of tumor is a tumor with the endocrine part at one end, the exocrine part at the other end, and an intermixed central zone [5]. The histopathological findings were confirmed by the same pathologist. Progression-free survival (PFS) refers to the time interval between operation and disease progression. Disease progression was defined according to RECIST guidelines [6]. Disease-specific survival (DSS) indicates the duration between the operation and the occurrence of disease-related death.

### Follow-up strategy

Follow-ups were conducted via an outpatient clinic and over telephone. The first clinic return visits were arranged at approximately 30 days after the operation. Follow-ups were performed every 3–6 months for the first 2 years and every 6–12 months thereafter. History acquisition, physical examination, and blood tests were performed at every follow-up. Enhanced CT or MRI was first executed 3 months after the surgery and every 6 months thereafter. If evidence of progression was found, frequent clinical and imaging assessments were recommended every 2–3 months.

### Statistical analysis

All statistical analyses were performed using SPSS software version 20.0 (SPSS, Chicago, IL, USA). Categorical variables were expressed as frequencies and percentages, whereas continuous variables were expressed as the median (range) or mean (s. d.) after the test of normality. Survival time (PFS or DSS) was estimated using the Kaplan-Meier method. Cox regression analysis was used to identify the predictive relationships between variables and survival. *P* < 0.05 was considered statistically significant.

## Results

### Clinicopathological features and survival data of the present cohort

A total of 2053 patients with pancreatic tumors were reviewed during the study interval, seven (0.34%) of these patients were pathologically confirmed to have pMANEC and thus examined. The median age of the patients was 46 years (range, 35–68 years). No obvious gender difference was observed (male: female = 3: 4). All but one patient complained of abdominal pain at admission (6/7). None of the seven cases was functional.

Five pancreatoduodenectomies and one distal pancreatectomy were performed. One patient did not receive an operation because the tumor was unresectable. Among the six resected cases, five had R0 resections, and one had R1 resection (retroperitoneal margin positive). The median operation time was 300 min (range, 285–530 min), and the median EBL was 600 ml (range, 400–1200 ml). Three of the six surgical procedures required perioperative blood transfusions. Regarding POPF, one patient had a Grade B fistulae (1/6). No Grade C fistulae occurred. Two incidences of Grade II-IV complications were identified according to Clavien-Dindo classification [7]. One patient suffered from delayed gastric emptying, which was resolved by fasting and decompression via an endoscopic nasojejunal feeding tube. One patient suffered from abdominal infection with *Klebsiella pneumonia* that was cured with antibiotics and adequate drainage. The median postoperative LOS was 21 days (range, 17–41 days). No death occurred within 30 days after surgery.

**Fig. 1:**
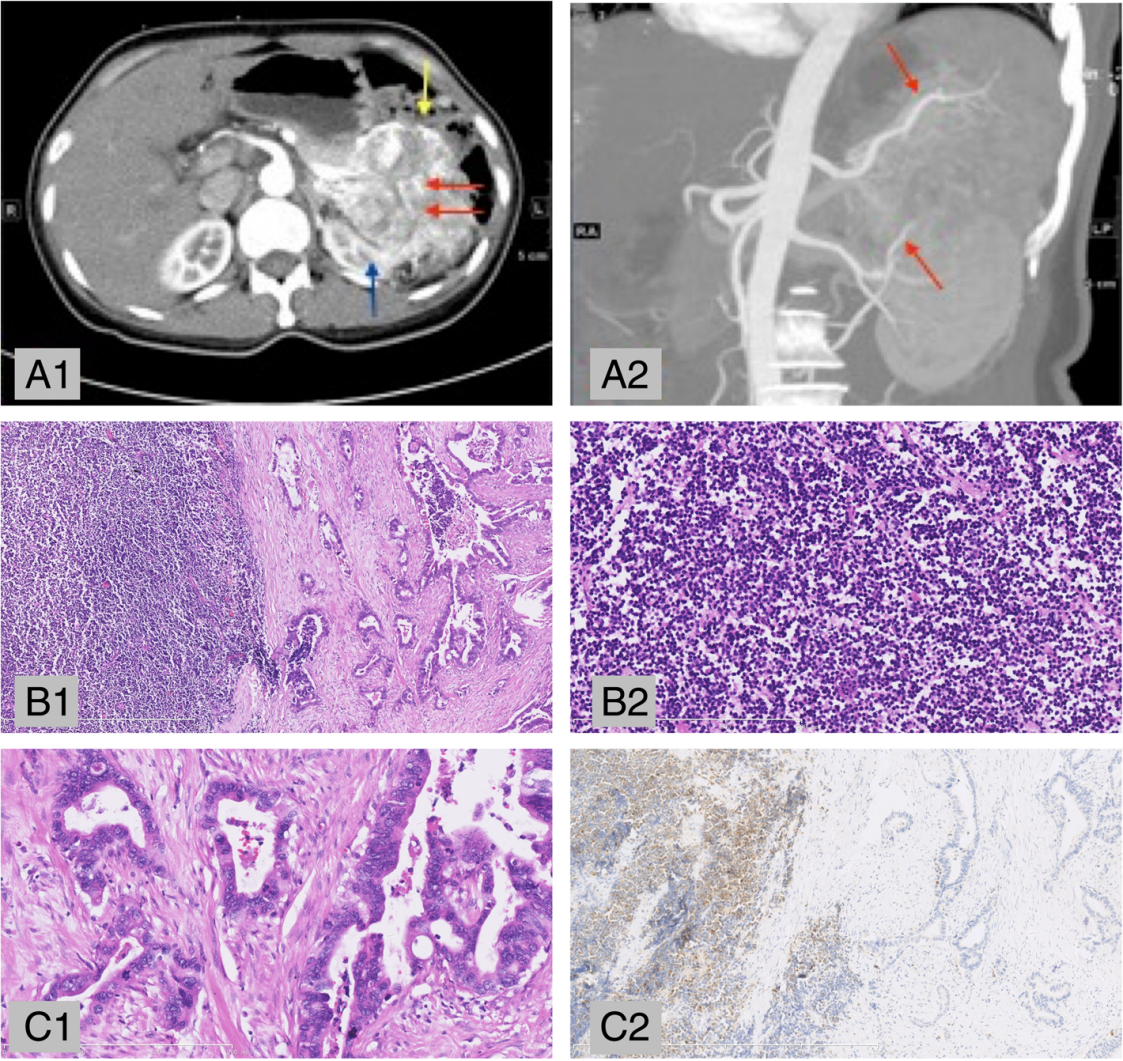
Imageological and pathological characteristics of pMANECs. Patient No. 24 (A1) A huge tumor located at the distal pancreas (red arrow) is heterogeneously enhanced in the arterial phase. The tumor caused a mass effect and presented a close relationship to the left kidney (blue arrow) and splenic flexure of the colon (yellow arrow). Neuroendocrine tumors were suspected preoperatively in this case. (A2) The coronal reconstruction showed that the tumor was fed mainly by branches of the splenic artery and inferior mesentery artery (red arrow). (B1) The primary lesion was composed of a poorly differentiated NEC component (left, small cell NEC) and a moderately differentiated adenocarcinoma component (right) (HE, 40×). (B2) The NEC component consists of diffuse tumor cells with prominent mitosis (HE, 200×). (C1) The adenocarcinoma component was composed of infiltrating duct-like structures and irregular neoplastic glands with intensive desmoplastic stromal reaction (HE, 200×). The typical neuroendocrine marker synaptophysin (C2). HE, hematoxylin and eosin staining; IHC, immunohistochemical staining

Pathologically, the specimens commonly presented with a yellowish or yellow-white color in our cohort. Microscopically, as illustrated in Fig. 1, the tumors composed of moderately to poorly differentiated adenocarcinoma and NEC. The ductal component consisted of large irregular ducts with columnar cells, while the endocrine component was formed by acini of small- to medium-sized cells with a solid or bridge shape. Metastatic lymph nodes could host either exocrine or endocrine components. As shown in Table 1, the neuroendocrine component of most cases was classified as G 2(4/6), and the rest were G 3(2/6) according to the Ki-67 index (Zhongshan Goldenbridge, China) and the mitotic rate. Immunohistochemical (IHC) staining showed that synaptophysin (Leica, Germany) and CD56 (Leica, Germany) were all positive in available cases for the NEC component, while chromogranin (Zhongshan Goldenbridge, China) was negative in two cases. CK19 (Dako, Denmark) was positive in the two detected cases for ductal component.

**Table 1:** Main histopathological details of surgically resected cases in this study

No.	CgA	Syn	CD56	CK19	NEC:PDCA	NEC Ki-67	PDAC Ki-67	NEC mitosis (10 HPF)	NEC grade
1	+	+	NA	NA	60%:40%	20%	15%	17	G2
2	+	+	+	NA	60%:40%	8%	25%	5	G2
3	+	+	+	NA	70%:30%	10%	30%	2	G2
4	+	+	NA	NA	70%:30%	10%	30%	8	G2
5	–	+	+	+	70%:30%	60%	30%	53	G3
6	–	+	NA	+	NA	60%	NA	NA	G3

*CgA* Chromogranin, *Syn* Synaptophysin, *NEC* Neuroendocrine carcinoma, *PDAC* Pancreatic ductal adenocarcinoma, *HPF* High powered field, + Positive, − Negative, *NA* Not available. Anti-CgA (Zhongshan Goldenbridge, China). Anti-Syn (Leica, Germany). Anti-CD56 (Leica, Germany). Anti-CK19 (Dako, Denmark). Anti-Ki-67 (Zhongshan Goldenbridge, China). The sixth case was operated in other hospital and transferred to our department after liver metastasis, so the information was based on the original report

One case was lost to follow-up. Five events of disease progression were detected, and the median PFS was 7.5 months (range, 2–36 months). Four disease-related deaths were observed. The media DSS was 15 months (range, 6–36 months).

### Total analysis combined with cases reported in the literature

Along with the twenty-one previously reported cases, the total statistical analysis included twenty-eight patients with pMANECs. Details were summarized in Table 2. The median age of patients at presentation was 59.5 years (range, 29–75 years). There was a strong male predominance (18/28, 64.3%). The most common manifestation was upper abdominal pain (15/28, 53.6%), followed by jaundice (10/28, 35.7%). Six (21.4%) patients presented asymptomatically when diagnosed. Taking symptoms and blood tests into consideration, the majority of tumors were non-functional (25/28, 89.3%). The rest were clinically functional (one gastrinoma, one glucagonoma and one VIPoma). As the most commonly described tumor marker, the expression of carbohydrate antigen 19–9 (CA 19–9) was elevated in 32.1% of the cases but presented normal in 35.7% of the cases.

**Table 2:** All cases of pancreatic mixed adenoneuroendocrine carcinoma involved in this study

No.	Reference	Age(year)	Sex	Presentation	Function	CA199 (U/ml)	Location	Treatment	Size (cm)	LN	Liver metastasis	Follow-up (month)
1	Eusebi (1981) [8]	65	M	Jaundice	NF	NA	H	PD	6.0	NA	Y	NA
2	Reid (1982) [9]	29	F	AP,WL,Jaundice	NF	NA	H	PD	NA	N	N	Alive (24)
3	Reid (1982) [9]	50	M	AP	NF	NA	H	DP	NA	Y	N	Alive (8)
4	Ordonez (1988) [10]	62	F	Diarrhea	VIPoma	NA	H	Chemo	4.0	NA	Y	Died (13)
5	Kashiwabara(1991) [11]	48	M	None	NF	Normal	H	PD	1.9	N	N	NA
6	Laine (1992) [12]	59	M	AP, Jaundice	NF	NA	H	PD	6.0	N	N	Alive (24)
7	Hassan (1993) [13]	50	M	AP,WL	NF	NA	D	DP	19.0	Y	N	Died (10)
8	Morikane (1997) [14]	54	F	None	Glucagonoma	Normal	D	DP	1.0	N	N	Alive (36)
9	Terada (1999) [15]	62	M	ZES	Hypergastrinemia	Normal	D	DP*	1.0	N	N	Died(288)
10	Leteurtre (2000) [16]	74	M	Jaundice, WL	NF	127	H	PD	3.0	Y	N	NA
11	Chatelain (2002) [17]	72	F	None	NF	NA	D	DP	10.0	N	N	Alive (4)
12	Terada (2002) [18]	34	M	AP	NF	Normal	D	DP	0.5	N	N	NA
13	Ballas (2005) [19]	65	F	AP, nausea	NF	NA	D	DP	12.0	N	N	Alive (18)
14	Hashimoto (2005) [20]	75	M	Jaundice	NF	1289	H	PD	3.5	N	N	Died (6)
15	Carter (2008) [21]	58	F	Jaundice	NF	NA	H	PD	2.0	Y	N	Alive (3)
16	Brandi (2008) [22]	68	M	AP	NF	Normal	D	DP	4.0	N	N	Died (12)
17	Araki(2011) [23]	68	M	None	NF	Normal	H	PD	2.0	N	N	Alive (52)
18	Murata(2017) [24]	66	M	Jaundice	NF	95.6	H	PD	3.0	Y	N	Died(12)
19	Xenaki(2016) [25]	51	M	AP, Jaundice	NF	Normal	H	PD	1.5	N	N	Died(13)
20	Kaji(2016) [26]	60	M	AP	NF	Normal	H	Chemo	3.0	NA	Y	Alive(18)
21	Imaoka(2016) [27]	63	M	None	NF	51.1	H	PD†	2.0	Y	N	Died(6)
22	The Present case	65	F	AP, Jaundice	NF	Normal	H	PD	2.3	Y	N	Died(15)
23	The Present case	39	F	AP	NF	131	H	PD	2.3	Y	Y	Died(32)
24	The Present case	35	F	AP	NF	46.7	D	DP‡	6.0	N	N	Alive(36)
25	The Present case	68	M	None	NF	74.4	D	PD§	4.0	Y	N	Died(6)
26	The Present case	42	F	AP, Jaundice	NF	52.6	H	PD†	3.8	Y	N	Alive(11)
27	The Present case	48	M	AP	NF	Normal	H	PD	5.0	N	N	Alive(5)
28	The Present case	46	M	AP, Back pain	NF	2832	D	Chemo	3.0	NA	N	Died(5)
	Total Results	Median age	M	AP	Functional	Elevated	H	PD	Median size	Y	Y	Median OS
		59.5(29–75)	64.3%	53.6%	89.3%	32.1%	64.3%	57.1%	3.0(0.5–19.0)	35.7%	14.3%	12.5(3–288)

*LN* Lymph node, *AP* Abdominal pain, *WL* Weight loss, *VIP* Vasoactive intestinal peptide, *ZES* Zollinger-Ellison syndrome, *NF* Not functional, *NA* Not available, *H* Head of pancreas, *D* Distal pancreas, *PD* Pancreatoduodenectomy, *DP* Distal pancreaectomy with or without splenectomy, Chemo, chemotherapy, *Y* yes, *N* No, *OS* Overall survival. *Plus total gastrectomy; †Plus portal resection and reconstruction; ‡Plus left nephrectomy, splenectomy and resection of splenic flexure of colon; §A total pancreatoduodenectomy was performed

pMANECs occurred more frequently at the pancreatic head (64.3%) than at the body or tail (35.7%). Ten out of the 28 patients received endoscopic ultrasound-guided fine needle aspiration (EUS-FNA) before surgeries, among which only three patients were confirmed pMANEC preoperatively. Most patients (16/28, 57.1%) received pancreatoduodenectomies, and 32.1% (9/28) of patients underwent distal pancreatectomies. The remaining patients (3/28, 10.7%) did not undergo surgery because of locally unresectable lesions. The size of the primary tumor varied from 0.5 cm to 19.0 cm, with a median diameter of 3.0 cm. LN metastasis and liver metastasis were observed in 35.7% (10/28) and 14.3% (4/28) of patients, respectively.

### Survival and prognostic analysis

The median follow-up period of the cohort was 12.5 months, ranging from 3 to 288 months. Among the 24 cases that had supplied survival data, twelve patients were identified with disease-specific death, with a median DSS of 12.5 months (range, 3–288 months). One patient died two days after surgery, possibly due to operation-related complications (details not described in the literature).

**Fig. 2:**
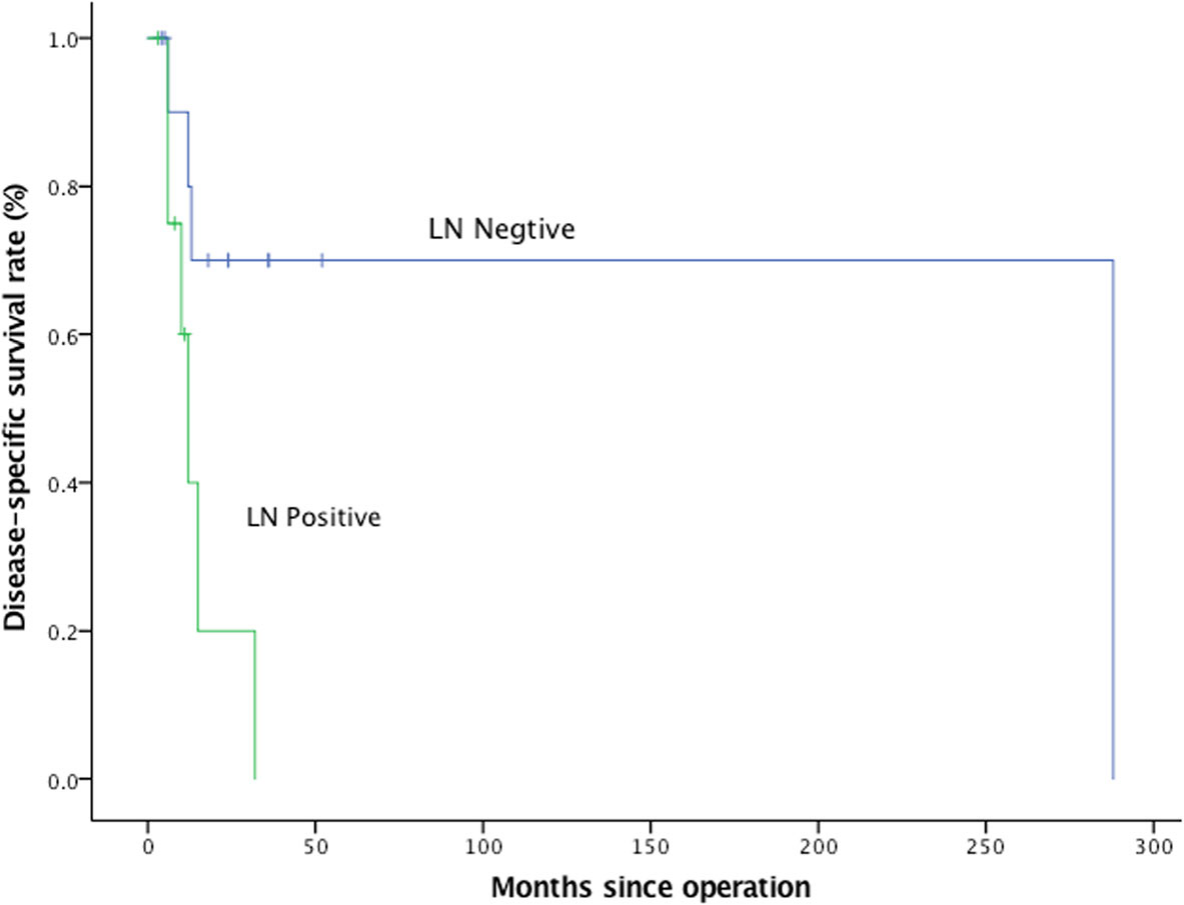
Median DSS according to lymph node metastasis. *P* = 0.015. The median DSS was 21 months in the LN negative arm, which was significantly longer than the 10 months of the LN positive arm

As shown in Fig. 2 and Table 3, LN metastasis (*P =* 0.015) had a significant prognostic effect on DSS, while age (*P =* 0.414), sex (*P =* 0.125), tumor size (*P =* 0.392), location (*P =* 0.913), functional status (*P =* 0.313), CA19–9 expression level (*P =* 0.608) and liver metastasis (*P =* 0.935) were not statistically significant predictors of DSS.

**Table 3:** Prognostic analysis of variables potentially associated with disease-specific survival for pancreatic mixed adenoneuroendocrine carcinoma

Variable	Groups	Number	Event	*P* value
Age (years)	≤59.5	12	4	0.414
	> 59.5	12	8	
Sex	Male	14	9	0.125
	Female	10	3	
Location	Head	15	7	0.913
	Body and tail	9	5	
Size (cm)	≤3.0	11	7	0.392
	> 3.0	11	5	
	NA	2	–	
Functional	No	21	10	0.313
	Yes	3	2	
CA19–9	Normal	8	4	0.608
	Elevated	8	6	
	NA	8	–	
Treatment	Resected	21	10	1.000
	Not-resected	3	2	
Positive LN	No	12	4	0.015*
	Yes	9	6	
	NA	3	–	
Liver metastasis	No	21	10	0.935
	Yes	3	2	

Only twenty-four cases were analyzed, which had supplied survival data. NA, not available. *P<0.05

## Discussion

In this study, we analyzed seven archival pMANEC cases constituting 0.34% of the pancreatic tumors of the study period. To our knowledge, this was the largest cohort worldwide from a single institution.

Similar to PDAC, pMANEC tended to occur in the elderly and in men. Abdominal pain was the most common symptom of both PDAC and pMANEC, which might be caused by tumor-occupying effects [28]. Obstructive jaundice appeared less frequently in pMANEC patients than in PDAC patients (35.7% vs. 56% [29]), although both tumors developed more in the head of the pancreas. This difference indicated that pMANEC was less able to infiltrate the bile duct compared to PDAC. The tumor size of pMANECs in the present study showed a wide range, from 0.5 to 19.0 cm, revealing the heterogeneity of pMANECs. This variation of growth was more similar to that of pure pNETs rather than PDAC [30], which was another point to distinguish pMANEC from PDAC except the difference of jaundice. However, no significant relationship was found between tumor size (*P =* 0.392) and DSS. Pathologically, the high level of Ki-67 index (ranging 8–80% in our cohort) and predominance of nonfunctional status (89.3%) have revealed higher proliferation and poorer differentiation of pMANECs relative to that of pNETs.

As the most commonly used examination, enhanced CT scans of pMANECs have presented heterogeneous imaging features, which could mimic the hypovascularity of PDAC, the hypervascularity of pNET or the mixed-density of SPT. Ten out of 28 patients received preoperative EUS-FNA, among which only three patients were revealed to have pMANEC, consistent with the postoperative pathological reports. The low sensitivity of EUS-FNA has well reflected the heterogeneity of pMANEC and difficulty in its preoperative diagnosis. As the only approach to histological diagnosis before treatment, performing EUS-FNA from different angles to increase accuracy has been suggested [26].

According to the data of our institution, high surgical quality could be provided based on the satisfactory R0 resection ratio (5/6), acceptable operative time, EBL and postoperative complication rate. The median DSS was 15 months for pMANEC, which was longer than the 8.5 months of unresectable pNECs described by Yamaguchi T et al. [31] but shorter than the 23 months of resected pNECs with high-grade reported by Sven-Petter Haugvik et al. [32]. Compared to PDAC, the median DSS of pMANEC was longer than the 9–10 months for unresectable tumors described by Hidalgo M et al. [28] but less than the 25.9–26.9 months for resected tumors reported by Itchins M et al. [33] from Australia. Although the above disadvantage might be associated with partial liver metastasis in the present study, the poor survival rate embodied the invasiveness, high proliferation, and poor differentiation of pMANECs and strongly indicated its high malignancy.

As shown in Table 2, the rate of LN metastasis reached up to 35.7%. It seemed that pMANEC mainly spread through lymphatic pathways. In the subsequent prognostic analysis, LN metastasis (*P* = 0.015) was found to have significant prognostic effects on DSS, with a median survival time of 21 months in the LN negative arm versus 10 months in the LN positive arm. This result was consistent with that of PDAC [34, 35]. Oppositely, nodal status was found not to be associated with survival of pNETs reported by Bilimoria KY et al. [36] and Fischer L et al. [37].

Only three of the operated patients were confirmed to have liver metastases (12%, 3/25), which was much lower than the 22.5% of PDAC [38]. It could be inferred that pMANEC was more likely to be resected due to the lower distal metastases. However, no significant relationship was found between liver metastasis and DSS. One reason for the lack of detectable relationship was the small sample size due to the rarity of pMANEC, which was the most obvious limitation of this study.

## Conclusions

pMANEC is a highly malignant tumor with a poor prognosis. The biological behavior of pMANEC is more similar to PDAC except less obstructive jaundice and wider range in tumor size. Lymph node metastasis was found to be a negative prognostic factor of DSS based on the present study.

## Data Availability

The diseases related data has been almost totally displayed in the manuscript except the confidential part. The original data and materials are available from the corresponding author on reasonable request.

## Abbreviations

DSS: disease-specific survival
EBL: estimated blood loss
EUS-FNA: endoscopic ultrasound-guided fine needle aspiration
HPF: high-power fields
IHC: Immunohistochemistry
LN: lymph node
LOS: postoperative length of hospital stay
PDAC: pancreatic ductal adenocarcinoma
PFS: progression-free survival
pMANEC: pancreatic mixed adenoneuroendocrine carcinoma
pNET: pancreatic neuroendocrine tumor
POPF: postoperative pancreatic fistulae
SPT: solid pseudopapillary tumor
VIP: vasoactive intestinal peptide
